# Role of Hydrogen Sulfide in the Endocrine System

**DOI:** 10.3389/fendo.2021.704620

**Published:** 2021-07-16

**Authors:** Hao-Jie Chen, Ebenezeri Erasto Ngowi, Lei Qian, Tao Li, Yang-Zhe Qin, Jing-Jing Zhou, Ke Li, Xin-Ying Ji, Dong-Dong Wu

**Affiliations:** ^1^ School of Basic Medical Sciences, Henan University, Kaifeng, China; ^2^ Henan International Joint Laboratory for Nuclear Protein Regulation, Henan University, Kaifeng, China; ^3^ Department of Biological Sciences, Faculty of Science, Dar es Salaam University College of Education, Dar es Salaam, Tanzania; ^4^ Kaifeng Key Laboratory of Infection and Biological Safety, School of Basic Medical Sciences, Henan University, Kaifeng, China; ^5^ School of Stomatology, Henan University, Kaifeng, China

**Keywords:** hydrogen sulfide, hormone, endocrine system, hypothalamus, pancreas

## Abstract

Hydrogen sulfide (H_2_S), as one of the three known gaseous signal transduction molecules in organisms, has attracted a surging amount of attention. H_2_S is involved in a variety of physiological and pathological processes in the body, such as dilating blood vessels (regulating blood pressure), protecting tissue from ischemia-reperfusion injury, anti-inflammation, carcinogenesis, or inhibition of cancer, as well as acting on the hypothalamus and pancreas to regulate hormonal metabolism. The change of H_2_S concentration is related to a variety of endocrine disorders, and the change of hormone concentration also affects the synthesis of H_2_S. Understanding the effect of biosynthesis and the concentration of H_2_S on the endocrine system is useful to develop drugs for the treatment of hypertension, diabetes, and other diseases.

## Introduction

It was not until the 1990s when endogenous and relatively high concentrations of hydrogen sulfide (H_2_S) were found in the brains of mice, humans, and cattle (1–3), that people’s inherent perception of H_2_S as a toxic gas changed. Exogenous H_2_S is a strong neurotoxin with a sturdy stimulating effect on mucosa. Inhalation of a small amount of high concentration of H_2_S can be fatal in a short time. A low concentration of H_2_S can affect the eyes, respiratory system, and central nervous system. With the progress of the research, more and more functions of endogenous H_2_S have been clarified, including vasodilation and angiogenesis (4, 5), cell death (6), intracellular signal transduction (7), inflammatory regulation (8), and mitochondrial energetics (9). Abnormal synthesis and decomposition of H_2_S can lead to a variety of diseases, including but not limited to hypertension, obesity, diabetes, arteriosclerosis, and cancer.

The endocrine syndrome occurs when the secretion and/or structure of the endocrine gland or endocrine tissue itself is abnormal. It also includes the syndromes of abnormal hormone sources, abnormal hormone receptors, and physiological disorders caused by the abnormal metabolism of hormones or substances. In the endocrine system, H_2_S can act on the thyroid (10), adrenal gland (11), and gonad (12) through the hypothalamus-pituitary axis, as well as on the pancreas (13), thereby participating in the regulation of many hormones in the body, and the hypothalamus-pituitary axis can, in turn, regulate the production of H_2_S (14).

The purpose of this paper is to discuss the effect of H_2_S on the endocrine system, hoping to have a better understanding of this new gas signal transduction molecule.

## Synthesis and Metabolism of H_2_S in the Endocrine System

At present, three main known enzymes produce H_2_S in organisms: cystathionine β-synthase (CBS), cystathionine γ-lyase (CSE), and 3-mercaptopyruvate sulfurtransferase (3-MST) (15). The substrate of CBS and CSE is L-cysteine (16), while 3-MST can catalyze 3-mercaptopyruvate to produce H_2_S (17, 18). Recently, it has been discovered that a kind of human methanethiol oxidase-selenium binding protein 1 (SELENBP1) can convert methanethiol into H_2_0_2_, formaldehyde, and H_2_S. In adipocytes, H_2_S can be produced by SELENBP1 and is related to the expression of CBS, CSE, and 3-MST (19–21). But the amount of the enzyme that produces H_2_S varies in different tissues and organs. In endocrine glands and endocrine organs, the RNA and protein expression levels of CBS are the highest in the pancreas, especially in acinar cells (22). Moreover, the CBS is rarely distributed in other endocrine glands such as thyroid, parathyroid, adrenal gland, and pituitary gland (23); however, abnormally increased in thyroid carcinoma (10). The expression of CSE is the most abundant in the liver, but low in the thyroid, pancreas, testis, ovary, and other endocrine glands (24–26).3-MST is highly expressed in endocrine tissues (thyroid, parathyroid, adrenal), pancreas, gonad (testis, ovary) (27). H_2_S can affect the secretion of many hormones and participate in the occurrence and development of endocrine diseases (11), but the effect of H_2_S on the body may be biphasic (15), that is, the effect of too high or too low concentration is the opposite, so to ensure the normal physiological function of the body, it is necessary to maintain the concentration of H_2_S at an appropriate concentration. It is well known that H_2_S can be regulated in two ways: synthesis and consumption. In terms of synthesis, H_2_S is synthesized mainly through enzymatic and non-enzymatic pathways (such as reduction of sulfur-containing compounds) and, in a few cases, released by bound sulfur stored in cells (28). In the endocrine system, H_2_S is mainly produced by three enzymes that catalyze different substrates. In terms of consumption, H_2_S can remove protons from mitochondria and rapidly oxidize to thiosulfate, which is then converted to sulfite and eventually oxidized to sulfate (28). H_2_S can also accept the methyl of thiol-S-methyltransferase to constitute dimethylsulfide and methanethiol. Or metabolized by thiohemoglobin, a complex formed by H_2_S and methemoglobin (29). The half-life of H_2_S *in vivo* is very short (a few seconds to a few minutes) (30, 31). Nowadays, it mostly inhibits the activity of synthase or increases the donor *in vitro*, which brings great difficulties to the study of H_2_S.

## Biological Effects of H_2_S in the Endocrine System

H_2_S can protect endocrine organs and regulate hormone secretion through anti-oxidative stress and ion channel regulation.

### Antioxidant Stress

H_2_S protects cells from oxidative stress in two ways: (1) directly scavenging reactive oxygen species (ROS) and (2) up-regulating antioxidant defense. Excessive ROS can cause oxidative stress, resulting in DNA damage, protein misfolding, organelle lesion, and neuronal synaptic dysfunction (32). The main substances that produce ROS in cells are peroxide and nitric oxide (NO) (33). In the heart, H_2_S reduces lipid peroxidation by scavenging peroxides; directly scavenges nitrite peroxides in neuroblasts, and binds to NO-free radicals to reduce oxidative stress (34–36). H_2_S up-regulates antioxidant defense mainly by activating the nuclear-factor-E2-related factor-2 (Nrf2) pathway and increasing the amount of glutathione (GSH). Nrf2 is an antioxidant regulator which regulates the gene expression of a variety of enzymes used to reduce oxidative stress (37). GSH is the main substance of antioxidant stress in cells, and H_2_S can distribute GSH into mitochondria. The ROS produced in mitochondria can be scavenged by GSH (38).

### Regulated Ion Channels

K_ATP_ channel is the first ion channel target of H_2_S (4), and many reported physiological effects of H_2_S are mediated by this channel: (1) vasodilation induced by H_2_S depends on its opening of K_ATP_ channel in vascular smooth muscle cells; (2) H_2_S opens K_ATP_ channel in rat atrial and ventricular myocytes; (3) electrophysiological studies have shown that NaHS increases K_ATP_ channel currents in rat aortic and smooth muscle cells. (4) the opening of the K_ATP_ channel also supports the relaxing effect of H_2_S on colonic and ocular smooth muscle (39–44). The Ca^2+^ channel is another ion channel regulated by H_2_S, which is elucidated in many types of cells, including nerve cells, cardiomyocytes, and endothelial cells. It is involved in cardiac contraction, angiogenesis, inflammation, and sensory transmission, and its effect may be related to the source of Ca^2+^ (extracellular or endoplasmic reticulum) (45–47). We will discuss the effects of the K_ATP_ channel and Ca^2+^ channel on insulin secretion below.

## The Mechanism of H_2_S Participating in the Physiological Process of the Endocrine System

**Figure 1:**
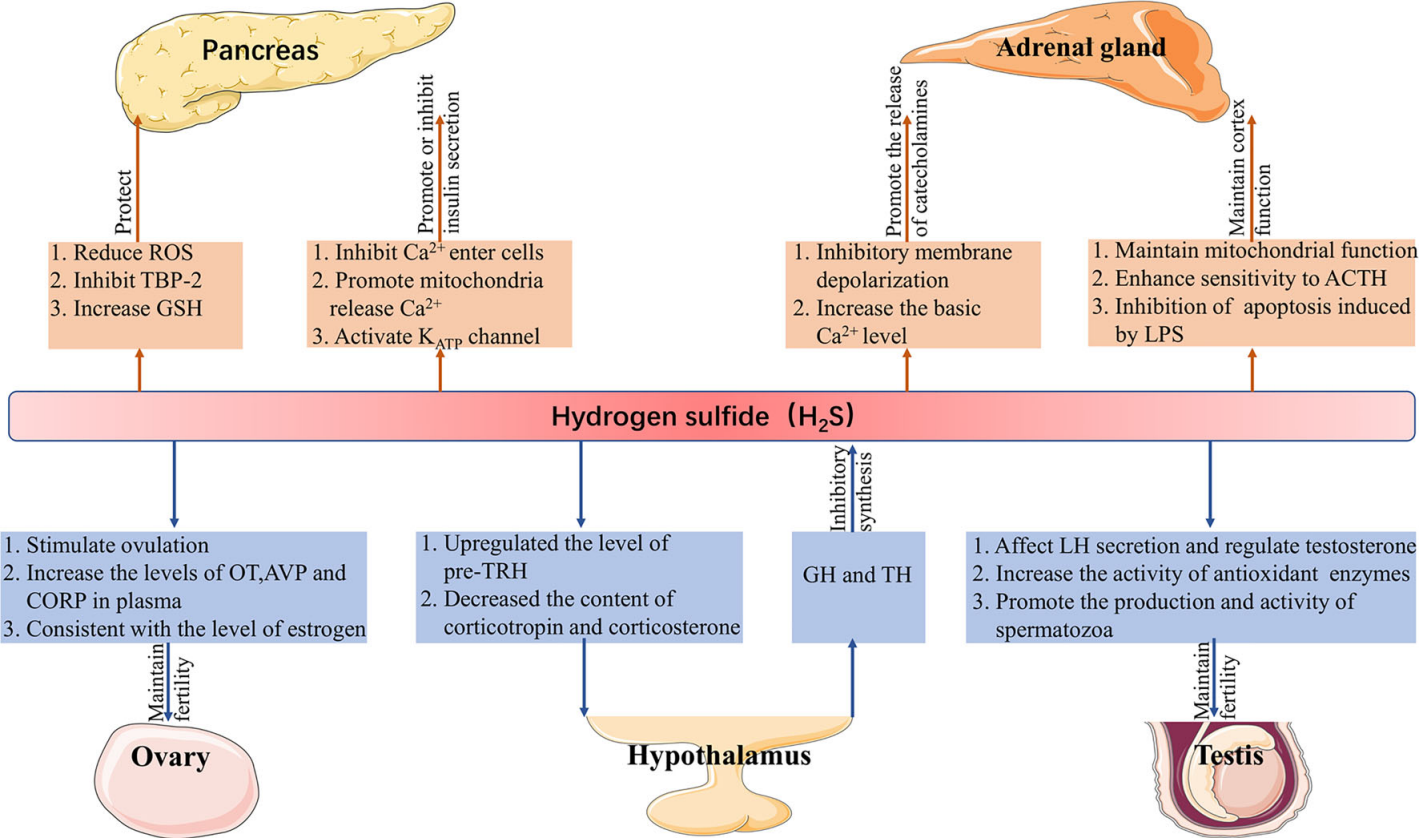
Function of H2S in the endocrine system. H2S, hydrogen sulfide; ROS, reactive oxygen species; TBP-2, thioredoxin binding protein-2; GSH, glutathione; ACTH, adrenocorticotropic hormone; LPS, lipopolysaccharide; LH, luteinizing hormone; pre-TRH, pre-thyrotropin-releasing hormone; GH, growth hormone; TH, thyroid hormone; OT, oxytocin; AVP, vasopressin; CORT, corticosterone.

H_2_S plays a role in the endocrine system to (1) protect pancreatic cells, regulate insulin secretion, (2) maintain the function of the adrenal cortex, promote the release of catecholamine, (3) maintain reproduction, and (4) regulate the secretion of pituitary hormones and are regulated by hormones (**Figure 1**).

### H_2_S in the Pancreas

H_2_S in the pancreas was first found in mice, and CBS and CSE were subsequently detected in these tissues (48, 49). The main roles of H_2_S in the pancreas are protecting pancreatic β cells and regulating insulin secretion.H_2_S may protect pancreatic β cells in the following three ways: (1) reduce the production of ROS; (2) inhibit the expression of thioredoxin binding protein-2-a redox protein associated with diabetes that promotes apoptosis; and (3) increase the content of GSH, all of which reduce the damage of oxidative stress (50–53). On the contrary, a high concentration of H_2_S induces apoptosis of pancreatic β cells (54). Insulin secretion is affected by many factors. It is known that the concentration and oscillation of Ca^2+^, K_ATP_ channel is related to H_2_S. H_2_S can not only inhibit the entry of Ca^2+^ from the plasma membrane into cells, to reduce insulin secretion, but also promote the release of Ca^2+^ in mitochondria and increase insulin secretion (55). It is well known that a high concentration of glucose can promote insulin secretion. In rat insulinoma cell line INS-1E, a high concentration of glucose (16mM) not only inhibits the activity of the K_ATP_ channel but also reduces the concentration of H_2_S, meanwhile H_2_S supplementation can activate the K_ATP_ channel (56). Since the activity of the K_ATP_ channel is also negatively correlated with insulin secretion (57). Therefore, it is unequivocal H_2_S can induce its effects on insulin secretion by utilizing this channel.

### The Role of H_2_S in the Adrenal Gland

In the adrenal gland, H_2_S can act on the adrenal cortex and chromaffin cells, respectively. For the adrenal cortex, inhibition of CBS/CSE can result in mitochondrial oxidative stress and dysfunction, while corticosterone response to the adrenocorticotropic hormone is weakened, which is reversed by morpholin-4-ium-methoxyphenyl-morpholino- phosphinodithioate (GYY4137, an H_2_S donor). Lipopolysaccharide (LPS) can lead to adrenal dysfunction induced by mitochondrial injury, and LPS inhibits the expression of CBS/CSE and the production of H_2_S in the adrenal gland, subsequent addition of GYY4137 inhibits apoptosis induced by LPS (58, 59). Calcium signaling plays a crucial role in the release of catecholamines (60). In mammals (mice and rats), the expression of CSE in chromaffin cells increased the oxygen sensitivity of the carotid body (61). Moreover, it has been proven that H_2_S can inhibit the depolarization of the membrane caused by IK^+^_Ca_ and induce the release of catecholamines in rat chromaffin cells (62). In calve chromaffin cells, the basal Ca^2+^ level increased in the presence of NaHS (H_2_S donor), thus promoting exocytosis (63). In fish (rainbow trout), electrical stimulation and acute hypoxia-induced increased secretion of catecholamines and H_2_S, followed by the addition of amino-oxyacetic acid (AOAA, CBS inhibitor), which significantly inhibited the production of H_2_S, while the effect of DL-Propargylglycine (PAG, CSE inhibitor) was not obvious (64).

### The Function of H_2_S in Gonads

The gonads mainly cover the testicles of men and the ovaries of women. Testes can secrete the male hormone testosterone, its main function is to promote the development of gonad and its accessory structure and the appearance of parasexual characteristics, as well as to promote protein synthesis (65). The expression of CBS and CSE are found in rat testes, but they are differentially expressed; CSE is mainly expressed in Sertoli cells and immature spermatogonia, while CBS is mainly distributed in Leydig cells, Sertoli cells, and germ cells (66). Sulfides in garlic regulate the secretion of testosterone by affecting the secretion of luteinizing hormone (LH), indicating that H_2_S may play an important role in the secretion of testosterone (67). Similarly, in rat testis, the provision of NaHS protected the testis from oxidative stress and inflammation induced by cisplatin and increased the activity of antioxidant enzymes (68). Subsequently, it has been found that the expression of CBS decreases in the spermatozoa of human asthenospermic patients, and AOAA inhibited the sperm motility, then the addition of GYY4137 weakens the effect of AOAA, suggesting that H_2_S could promote the production and activity of spermatozoa (69).

The decreased fertility of female offspring of CBS knockout mice suggests that H_2_S may have a greater effect on female fertility (70). In different mammals, H_2_S regulates female reproduction in many ways such as; the LH peak before ovulation in mice increases with the expression of H_2_S producing enzymes, the inhibition of CSE by hydroxylamine hydrochloride prevents ovulation, whereas, the subsequent addition of NaHS reverses the effect of the inhibitor, Na_2_S, an H_2_S donor, increases the plasma level of oxytocin, vasopressin, and corticosterone in rats; CBS increased the level of H_2_S in the uterine artery during sheep production, and the change of CBS protein expression was consistent with the estrous cycle and follicular estrogen level of sheep, so as the estrogen level of the human menstrual cycle (12, 71, 72).

### Interaction Between H_2_S and Hypothalamus-Pituitary Axis

Abundant CSE and CBS were found in the hypothalamus (73). In the paraventricular nucleus (PVN), the high expression of CBS upregulates the level of pre-thyrotropin-releasing hormone (pre-TRH) and decreases the content of corticotropin and corticosterone in blood (74). Interestingly, in hypothalamic explants, NaHS did not affect the physiological secretion of corticotropin-releasing hormone (CRH) but could inhibit the release of CRH stimulated by KCl (75). The results show that the hormones produced by the hypothalamus-pituitary axis also affect the synthesis of H_2_S. Thyroid hormone (TH) and growth hormone (GH) regulates the production of H_2_S in the liver through TH receptor β1 and GH receptor, respectively. In mechanism, TH inhibits the expression of CSE, while GH inhibits the production of H_2_S through substrate availability control by autophagy (14). However, another study showed that in hyperthyroidism rats, the level of H_2_S in the liver increased (76), which may be due to the different effects of different concentrations of TH on the H_2_S producing enzyme.

## Endocrine Diseases Involved in H_2_S

H_2_S can regulate glucose and fat metabolism through the pancreas, liver, adipose tissue, and skeletal muscle. In human thyroid and ovarian cancer, H_2_S appears to have a dual effect, which may be related to its concentration (or metabolic rate) in the cells. High concentrations of H_2_S can aggravate pancreatitis and induced lung injury, but protect osteoblasts, making it a potential drug for the prevention of osteoporosis.

### H_2_S and Type 2 Diabetes

Pancreas, liver, adipose tissue, and skeletal muscle are involved in the regulation of plasma glucose, the enzyme-producing H_2_S is expressed in all the above tissues (48, 77–79). H_2_S can manage plasma glucose levels by protecting pancreatic β cells and regulating insulin secretion (see section 4.1). H_2_S also seems to have the opposite effect on liver glucose uptake, glycogen storage, and gluconeogenesis. One point of view is that H_2_S promotes liver glycogenesis: in hepatocellular carcinoma cell line HepG2, NaHS inhibits the activity of glucokinase, and CSE overexpression reduces glycogen content, while CSE knockout increases glycogen content (80, 81). Another point of view is that H_2_S inhibits gluconeogenesis in the liver: the lack of CSE promotes gluconeogenesis in the liver, knockout CSE of HepG2 increases glucose production, while the addition of NaHS weakens the knockout effect of CSE (82). The role of H_2_S in regulating plasma glucose through adipose tissue also seems to be twofold. On the one hand, H_2_S increases glucose uptake and fat production in adipocytes, for example: (1) in 3T3L1 adipocytes, H_2_S improves high glucose-induced insulin resistance by up-regulating phosphatidylinositol 3,4,5-trisphosphate level, (2) in diabetic insulin resistance rats, NaHS and H_2_S gas solution improve insulin resistance by activating insulin receptors (IR), (3) CSE/H_2_S converts glucose into triglycerides in adipocytes through peroxisome proliferator-activated receptor γ (PPARγ) and stores them in cells, (4) H_2_S promotes adipogenesis in 3T3L1 cells by increasing the expression of fatty acid protein 4 (83–86). On the other hand, H_2_S plays a vital role in adipocyte insulin resistance mediated by tumor necrosis factor-α (TNF-α) (87). H_2_S improved insulin resistance of skeletal muscle by increasing the sensitivity of the IR-PI3K-Akt signal pathway. In mouse myoblast C_2_C_12_, the addition of NaHS increased glucose uptake (84, 88). The above results show that H_2_S reduces the level of plasma glucose by regulating numerous cellular markers.

### Effect of H_2_S on Thyroid Cancer

The effect of H_2_S on cancer is related to its concentration, that is, low concentration (or low production rate) and high concentration (or rapid production rate) can show opposite results (15).In thyroid cancer, two different H_2_S donors showed different results. Diallyl trisulfide (DATS), an H_2_S donor from garlic, has an inhibitory effect on both anaplastic and papillary thyroid cancer. In anaplastic cancer cell line 8505C, DATS induces the accumulation of ROS, which inhibits cell survival and increases apoptosis. In addition, it promotes cell DNA damage and arrests the cell cycle in the G2/M phase (89). In papillary carcinoma cell line KTC-1, treatment with diallyl trisulfide (DATS) activates NK-κB signaling pathway and increases the expression of NF-κB-dependent CSE, while H_2_S induced cell growth inhibition (90). While in another thyroid papillary carcinoma cell line BCPAP, DATS blocks the cell cycle in G0/G1 phase and promotes apoptosis of thyroid cancer cells by inducing mitochondrial apoptosis and activating the MAPK signal pathway (91). When NaHS is used as an H_2_S donor, a low concentration (≤50uM) of NaHS promotes the proliferation, migration, and invasion of thyroid cancer cells *in vitro*, while a high concentration (200uM) has the opposite result. In BALB/C nude mice with axillary tumorigenesis, the tumor mass increases, the doubling time shortens and the inhibition rate decreases in low concentration (≤2.8mg/kg/day) of NaHS, while in high concentration (≥5.6 mg/kg/day) the effect is reversed (92).

### H_2_S Promotes Acute Pancreatitis and Its Induced Lung Injury

**Figure 2:**
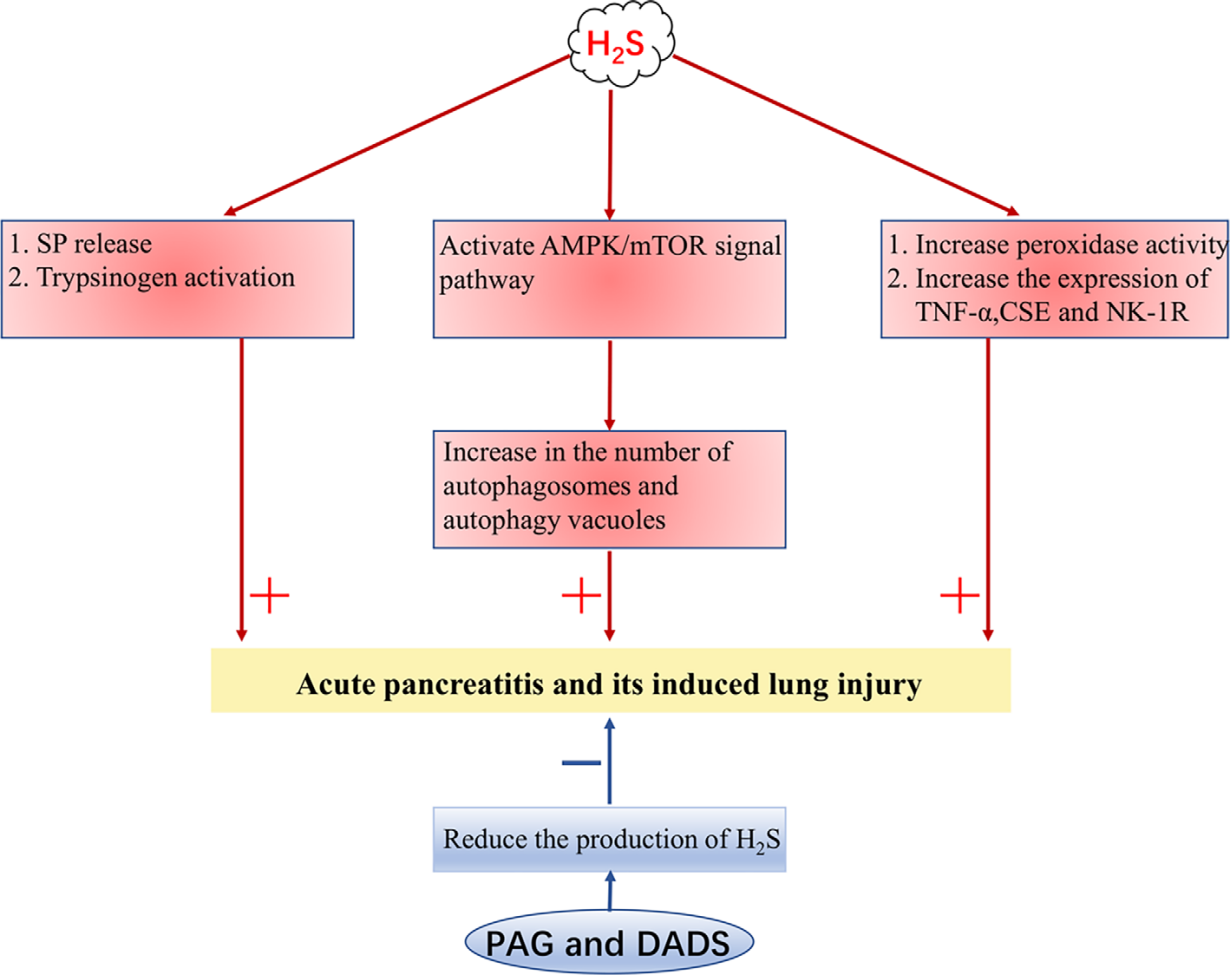
H_2_S aggravates acute pancreatitis. H_2_S, hydrogen sulfide; SP, substance P; TNF-α, tumor necrosis factor-α; NK-1R, neurokinin-1 receptor; CSE, cystathionine γ-lyase; PAG, DL-Propargylglycine; DADS, diallyl disulfide. + indicates aggravate, - indicates lighten.

In rats and mice with acute pancreatitis (AP), it has been found that the concentration of serum H_2_S is increased and the expressions of CSE and TNF-α are also increased in the lungs. The pro-inflammatory effect of H_2_S may be related to the release of substance P (SP) and the activation of trypsinogen, while the release of SP is mediated by neurokinin-1 receptor (NK-1R) in pancreatic acini (48, 93, 94). In addition, the number of autophagosomes and autophagy vacuoles increases in AP, which is positively correlated with the level of H_2_S, which associates with the activation of the AMPK/mTOR signal pathway (95). The addition of PAG decreases the activity of peroxidase in the lung and pancreas, the expression of TNF-α, CSE, and NK-1R, and the concentration of H_2_S in serum (93, 94, 96). Diallyl disulfide in garlic can also trigger an anti-inflammatory effect in the pancreas and lung by reducing the expression of TNF-α, CSE, NK-1R, and the production of H_2_S (97). To sum up, H_2_S can aggravate AP and its induced lung injury in many ways, and inhibiting the production of H_2_S may be a potential method for the treatment of AP (**Figure 2**).

### H_2_S and Obesity

H_2_S can increase or decrease fat synthesis in many ways. It is described previously by regulating insulin sensitivity in adipocytes. The expression of H_2_S in different tissues can jointly regulate the formation of fat. Overexpression of CBS in PVN reduces obesity and insulin resistance induced by a high-fat diet (HFD). Mechanistically, leptin up-regulates CBS expression through FOXO3a, promotes pre-TRH production by inhibiting the mTOR pathway, and then increases TH production and decreases corticosterone production, thus reducing fat deposition and improving insulin resistance induced by FHD (74). However, in mouse adipocytes, H_2_S promotes adipogenesis by activating PPARγ, where when CSE is deficient, mice show resistance to FDH-induced obesity (98). Perivascular adipose tissue provides protection and support for blood vessels. In obese patients, the secretion of pro-inflammatory macrophages increases, resulting in increased consumption of H_2_S in vascular endothelium and smooth muscle, which directly damages endothelium-dependent vasodilation, and results in a loss of pulmonary vein function (99).

### Uncertainty of the Effect of H_2_S on Ovarian Cancer

The expression of CBS is increased in ovarian cancer cell line A2780. After down-regulation of CBS in ovarian cancer cell line A2780, the content of GSH decreased, which aggravated the cascade of apoptosis triggered by oxidative stress and enhanced the sensitivity to cisplatin. In addition, mitochondrial function is inhibited and ATP synthesis decreases after down-regulation of CBS (100). Moreover, selenium-containing chrysin, as a compound with anticancer and antioxidant effects, shows an antitumor effect in many ovarian cancer cell lines, and its inhibitory effect is achieved by inhibiting the expression of CBS and promoting the transformation of GSH (101). However, another data shows that GYY4137 promotes the release of Ca^2+^ from the endoplasmic reticulum of A2780, induces endoplasmic reticulum stress, and drives apoptosis, while the content of GSH is decreased (102).

### H_2_S Inhibits the Development of Osteoporosis

Osteoporosis occurs in postmenopausal women and can also occur in patients with specific hormone secretion disorders or patients treated with glucocorticoid drugs. Endogenous H_2_S protects osteoblasts MC3T3-E1 from cytotoxicity induced by ROS. NaHS safeguards osteoblasts from oxidative stress-induced cell injury and inhibition of proliferation and differentiation through the MAPK signal pathway (103). Therefore, H_2_S may be used as a drug to prevent osteoporosis.

## Conclusions

The hypothalamic-pituitary-target organ axis is a complex biological structure, which plays an important role in endocrine regulation. H_2_S is distributed in multiple sites in the hypothalamus, pituitary-target organ, making it an important molecule in regulating hormone secretion, which is not only dual but also bi-directional, that is, H_2_S not only promotes the secretion of certain hormones but can also inhibit them. The complex effect of H_2_S on the endocrine system may be caused by its action on different organs. Current studies mainly focus on the effect of H_2_S on a single organ, the role of feedback regulation is unclear, which brings a difficult problem to explore the mechanism of action of H_2_S in the endocrine system.

There’s a growing body of evidence that H_2_S plays an important role in type 2 diabetes, but its effect on insulin secretion and insulin target organs has not been consensus, which may be related to its concentration and production rate in the target organs. Similarly, H_2_S is also involved in the occurrence and development of endocrine organ tumors, and its role in thyroid cancer is related to its concentration. Since the exact concentration of H_2_S in cells is difficult to detect, the effect of a specific concentration of H_2_S on endocrine organs remains to be further explained.

Although there are still many problems to be solved, existing studies have shown that H_2_S plays a key role in maintaining the function of endocrine organs and hormone secretion. Therefore, the regulation of H_2_S production may be a potential treatment for endocrine diseases.

## Author Contributions

X-YJ and D-DW contributed to conception and design of the study. H-JC looked at the data and wrote the first draft. EN, LQ, TL, Y-ZQ, J-JZ, and KL wrote sections of the manuscript. D-DW and EN reviewed and revised the first draft. All authors contributed to the article and approved the submitted version.

## Funding

This work was supported by grants from the National Natural Science Foundation of China (Nos. 81802718, 81670088, U1504817), the Foundation of Science & Technology Department of Henan Province, China (Nos.202102310480, 182102310335, 192102310151), the Training Program for Young Backbone Teachers of Institutions of Higher Learning in Henan Province, China (No. 2020GGJS038), and the Science Foundation for Young Talents of Henan University College of Medicine, China (No. 2019013).

## Conflict of Interest

The authors declare that the research was conducted in the absence of any commercial or financial relationships that could be construed as a potential conflict of interest.
